# Berberine HCl and diacerein loaded dual delivery transferosomes: Formulation and optimization using Box-Behnken design

**DOI:** 10.5599/admet.2268

**Published:** 2024-04-29

**Authors:** Siddharth Singh, Rajendra Awasthi

**Affiliations:** Department of Pharmaceutical Sciences, School of Health Sciences & Technology, UPES, Dehradun, Uttarakhand, India

**Keywords:** Inflammatory cytokines, pro-inflammatory, tumour necrosis factor, psoriasis, ultraflexible vesicles

## Abstract

**Introduction:**

Berberine is a poorly water-soluble alkaloid compound showing significant anti-inflammatory characteristics. It reduces the levels of pro-inflammatory and inflammatory cytokines, including tumour necrosis factor (TNF-α, IFN-γ) and interleukin (IL-23, IL-12, and IL-23). Diacerein significantly reduces the splenomegaly associated with psoriasis. It downregulates the production of TNF-α and IL-12.

**Method:**

This study reported the development of transferosomes containing berberine HCl and diacerein using a film hydration method followed by optimization using a Box-Behnken design. Sodium deoxycholate was used as an edge activator. The impact of independent variables (amount of phosphatidylcholine, amount of edge activator, and sonication cycles) on dependent variables (particle size and entrapment efficiency) was examined. The optimized formulation was characterized for polydispersity index, vesicle size, entrapment efficiency, ζ potential, spectral analysis like Fourier transform infrared, thermal analysis, X-ray diffraction, deformability, transmission electron microscopy, antioxidant assay, *in-vitro* release, and *ex-vivo* skin permeation studies.

**Results:**

The optimized formulation had a particle size of 110.90±2.8 nm with high entrapment efficiency (89.50±1.5 of berberine HCl and 91.23±1.8 of diacerein). Deformability, polydispersity index, ζ potential, and antioxidant activity of the optimized formulation were 2.44, 0.296, -13.3, and 38.36 %, respectively. Optimized transferosomes exhibited 82.093±0.81 % and 85.02±3.81 % release of berberine HCl and diacerein after 24 h of dissolution study. The transdermal flux of optimized formulation was 0.0224 μg cm^-2^ h^-1^ (2.24 cm h^-1^ permeation coefficient) and 0.0462 μg cm^-2^ h^-1^ (4.62 cm h^-1^ permeation coefficient), respectively, for berberine HCl and diacerein. Raman analysis of treated pig skin confirmed that the transferosomes can permeate the skin. No change in the skin condition or irritation was observed in BALB/c mice. Formulation stored at 4 and 25±2 °C / 60±5 % relative humidity was stable for 3 months.

**Conclusions:**

Thus, the results demonstrated successful optimization of the transferosomes for the efficient topical delivery of berberine HCl and diacerein in the effective management of psoriasis.

## Introduction

Psoriasis is a chronic autoimmune condition that affects an estimated 1 to 3 % of the worldwide population. It is characterized by an anomalous proliferation of keratinocytes, followed by an infiltration of cells associated with inflammation. The average duration of the maturation process of keratinocytes in healthy skin is about 30 days. However, people with psoriasis have an accelerated process, completing it within a substantially shorter timeframe of 5-7 days [1,2]. Psoriasis is subject to multiple etiological factors, including, but not limited to, trauma, stress, hereditary genetic predisposition, and other determinants that exert an influence on the physiological functioning of keratinocytes. The appearance of white scaly plaques, accompanied by itching and pain, has the potential to greatly intensify patient distress and perhaps result in the development of serious cutaneous diseases [3]. In addition to cutaneous morphological concerns, individuals diagnosed with psoriasis also have comorbidities such as depression and anxiety, which can contribute to the development of suicidal ideas and behaviours. A variety of therapeutic interventions are available for the management of psoriasis. However, it is crucial to note that they have limitations and unfavourable outcomes, which make it difficult to use them in clinical settings [4-7]. Topical drug delivery focuses on localized treatment by applying drugs directly to the skin's surface, allowing for targeted action at the application site while reducing systemic side effects. This method is especially useful for treating skin disorders like psoriasis, as it can improve treatment efficacy and patient compliance [8].

Promising developments in nanotechnology have led to the development of nanoparticulate drug carriers, which hold immense promise for treating a wide range of diseases. Nanomedicines are promising therapeutics for topical drug delivery. Transferosomes are a unique type of vesicle consisting of concentric layers made up of phosphatidylcholine (C18), cholesterol, and an edge activator and possess either an aqueous or ethanolic core [9]. Phosphatidylcholine is the predominant phospholipid found in the cell membranes of eukaryotic organisms. Consequently, it demonstrates a notable degree of skin tolerance, thereby decreasing the probability of negative effects such as hypersensitivity reactions that make them suitable for delivering drugs to sensitive areas [2]. Edge activators possess exceptional deformability and flexibility, enabling them to traverse the stratum corneum effortlessly without encountering issues such as fracturing or leakage. This enhances the delivery of drugs to target tissues. The flexible nature of transferosomes allows them to encapsulate a higher drug payload, leading to improved bioavailability as more of the drug reaches the target site. Transferosomes can adapt to the skin's topography, reducing irritation and minimizing the potential for skin damage. Transferosomes can encapsulate both lipophilic and hydrophilic drugs, providing versatility in formulation. The lipid composition of transferosomes contributes to their stability, ensuring the integrity of the encapsulated drug during storage and application. This leads to a longer shelf life for formulated products. The topical application of transferosomes is non-invasive, offering patients convenience and potentially improving compliance. This is particularly beneficial for conditions where systemic drug delivery is not required. Compared to liposomes and niosomes, they exhibit a significant degree of stability in water-based solutions for three months [10,11]. Compared to liposomes, tacrolimus-loaded transferosomes had a longer retention time (52.58±3.62 h) and better skin permeation (15.44±1.79 h). Compared to liposomes, optimised transferosomes exhibited superior penetration (15.44±1.79 h) and residence time (52.58±3.62 h) in rat skin. Transferosomes minimize ear edema while increasing transdermal flux [12]. Thin film hydration approach produced fluocinolone acetonide-loaded transferosomes with high transdermal flow, enhanced cytotoxicity, and antiproliferative efficacy against imiquimod-stimulated HaCaT cells. Transferosomes decreased IL-6 and TNF-α levels and increased cellular absorption [13].

Berberine, a compound present in Berberis vulgaris, is an alkaloid compound showing significant anti-inflammatory characteristics. It displays a distinct yellow crystalline morphology and exhibits significant water insolubility. Berberine suppresses the JAK-STAT pathway in plaque psoriasis. It decreases the levels of pro-inflammatory and inflammatory cytokines, including tumour necrosis factor-alpha (TNF-α, IFN-γ) and interleukin (IL-23, IL-12, and IL-23) [14,15]. Diacerein has been licensed by the European Medicines Agency and the US Food and Drug Administration for the treatment of rheumatoid arthritis. Diacerein inhibits the synthesis of IL-12 and TNF-α. Diacerein significantly reduces the splenomegaly associated with psoriasis [16,17].

The main aim of this study is to develop transferosomes containing controlled delivery of berberine hydrochloride and diacerein for better management of psoriasis. To achieve this objective, the thin film hydration method was used to formulate transferosomes, which were then tested for particle and ζ potential, drug loading, encapsulation efficiency, morphology, degree of deformability, drug release, and *ex vivo* skin permeation.

## Experimental

### Materials

Berberine HCl and diacerein were purchased from Yarrow Chem Products, Mumbai, India. Methanol and chloroform were obtained from Sigma Aldrich (MA, United States). Sodium deoxycholate was purchased from SD Fine Chemicals (Mumbai, India). Phosphatidylcholine, sodium hydroxide, and potassium dihydrogen phosphate were purchased from SRL Chemicals (Mumbai, India). Dialysis membrane (molecular weight cut off 12000 Da) was procured from Hi-Media Laboratory (Mumbai, India).

### Preparation of berberine HCl-diacerein loaded dual delivery transferosomes

Utilizing a digital rotatory evaporator (DLAB RE 100-Pro, Thermo Life Sciences, Mumbai, India), berberine HCl and diacerein-loaded transferosomes were prepared by the thin film hydration method. Phosphatidylcholine, sodium deoxycholate, and diacerein were weighed and dissolved in a chloroform and methanol mixture (2:1 ratio). The flask was rotated at 100 rpm and 47 °C for 30 minutes to form a thin film. The flask was kept in a desiccator for 24 h. Berberine HCl was dissolved in the phosphate buffer (PBS pH 7.4) and used for film hydration at 37 °C for 1 h (Table 1). To obtain drug-loaded transferosomes, the mixture underwent sonication by a probe sonicator at three different intervals (1, 3, and 5 min) with a pulse of 20 seconds on and off to obtain the berberine HCl-diacerein loaded-loaded transferosomes [18]. Transferosomes were kept in a deep freezer (ULT-185, REMI, India) at -20 °C for 24 h. The product was lyophilized at -45 °C under a suitable vacuum for 24 h (Alpha 2-4 LSCplus, Christ, Germany) [19].

**Table 1.** Box-Behnken design with the levels of independent variables used to formulate berberine HCl and diacerein-loaded dual delivery transferosomes.

Formulation code	Independent variable		
	*A* (amount of phosphatidylcholine)	*B* (amount of sodium deoxycholate)	*C* (sonication cycle time)
DDT-1	1	-1	0
DDT-2	0	-1	-1
DDT-3	0	1	1
DDT-4	0	-1	1
DDT-5	-1	1	0
DDT-6	1	1	0
DDT-7	1	0	-1
DDT-8	-1	0	-1
DDT-9	0	1	-1
DDT-10	0	0	0
DDT-11	-1	0	1
DDT-12	1	0	1
DDT-13	-1	-1	0
Codes for different levels of independent variables	Values for independent variables		
	Amount of phosphatidylcholine, mg	Amount of sodium deoxycholate, mg	Sonication cycle time, min
-1	75	5	1
-0.5	80	10	2
0	85	15	3
0.5	90	20	4
+1	95	25	5

Dependent variables: *Y*_1_ - Particle size (minimum); *Y*_2_ - Entrapment efficiency of berberine HCl and *Y*_3_ - Entrapment efficiency of diacerein (maximum)

### Experimental design for optimization of dual drug-loaded transferosomes

To find the best dual drug-loaded nanoparticles, a Box-Behnken design (trial version, Design Expert software, StatEase®, Minneapolis, Minnesota) was used to investigate how independent variables (*A* - amount of phosphatidylcholine, *B* - amount of edge activator and *C* - sonication cycles time) affected dependent variables (particle size and entrapment efficiency). Levels of independent variables were decided according to preliminary trials done. The effect of independent factors on dependent factors was optimized by using a 3-level factorial design. Statistical tests, including One-way analysis of variance (ANOVA) followed by Bonferroni multiple comparison test, goodness-of-fit tests, and regression coefficient (*r*^2^) tests, were used to ascertain the relevance of the applied model.

### Mathematical model

In order to accurately assess the effects of independent factors on dependent variables, a nonlinear quadratic polynomial model was developed using the Design Expert software trial version (Stat-Ease, Inc., Minneapolis, MN). The nonlinear quadratic polynomial model was developed considering the replicated center points and points placed on the multidimensional cube's midpoints (Equation 1). To optimize the formulation, the center point region of the multidimensional cube was deemed significant for assessing the effect of formulation variables.


(1)

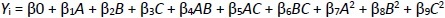



where *Y*_i_ is the dependent variable related to independent variables *A, B* and *C* (Table 1). The impact of the independent variables was assessed on the dependent factors *Y*_1_ (particle size), *Y*_2_ (entrapment efficiency of berberine HCl), and *Y*_3_ (entrapment efficiency of diacerein). The statistical design was validated by the significant responses depicted by the polynomial equations, and the model optimization and validation were considered by determining the ANOVA (*F*-value) and *p* < 0.05. Moreover, the counterplots and 3D response surface were plotted to establish the relationship and interaction among the variables and responses. The difference between predicted and actual values was determined by optimizing extra checkpoints. As per the number of runs received by the Box-Behnken design, the formulations were prepared and evaluated [12].

### Characterization of dual delivery transferosomes

#### Drug loading and encapsulation efficiency

For the estimation of drug loading and entrapment efficiency, 5 mL of sample was dispersed into 5 mL of PBS (pH 7.4) solution and centrifuged (REMI C-24 PLUS, Mumbai, India) at 10000 rpm for 30 min at 4 °C. After centrifugation, the supernatant was collected. The free berberine HCl and diacerein were analyzed using a UV spectrophotometer (UV-1900 Shimadzu, Japan) at 340 and 258 nm, respectively. The drug loading and entrapment efficiency were calculated using Equations (2) and (3) [18,20]:


(2)

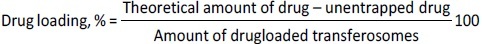




(3)

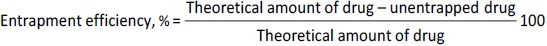



#### Determination of vesicle size, polydispersity index, and zeta potential

The prepared transferosomes were diluted with PBS (pH 7.4) to alleviate the opalescence in the solution. Diluted transferosomal solution (20 μL) was transferred into the cuvette and examined for particle size and polydispersity index (PDI) at room temperature using a Malvern particle size analyzer (EN1690, Malvern Instruments, United Kingdom). The average particle size and PDI values were taken in triplicate at a dissipating angle 90°. Laser doppler velocimetry was recorded to estimate the ζ potential values using a Zetasizer (Litesizer 500, AntonPaar, Austria) [19].

#### Fourier transform infrared analysis

Fourier transform infrared (FTIR) study of dried berberine HCl, diacerein, phosphatidylcholine, sodium deoxycholate, and drug-loaded optimized transferosomes (formulation DDT-10) was carried out by the KBr disk method using a Fourier transform infrared spectrophotometer (Frontier FT-IR/FIR, PerkinElmer, Norwalk, USA). The pellets were prepared by mixing samples and KBr at a 1:10 ratio. The pellets were kept in the sample holder and scanned from 4000 to 400 cm^-1^ [21].

#### X-ray diffraction analysis

To study the lattice structure, X-ray diffraction (XRD) study of berberine HCl, diacerein, phosphatidylcholine, sodium deoxycholate, and drug-loaded optimized transferosomes (formulation DDT-10) was carried out by a X-ray powder diffractometer (D8 Advance Eco, Bruker, United States) using Cu Kα radiation with a 40 kV voltage, 15.4 nm wavelength, and 200 mA current. Samples were scanned within the 2*θ* range of 0-90° at a step size of 0.02° [22].

#### Thermogravimetric analysis

Thermal gravimetric analysis (TGA-50, Shimadzu, Japan) determined the thermal degradation properties of berberine HCl, diacerein, phosphatidylcholine, sodium deoxycholate, and drug-loaded transferosomes (formulation DDT-10). The sample (5 mg) was kept on the crucible under insert conditions, and the thermosgrams were obtained between 30 to 900 °C [20].

#### Morphological characterization

High-resolution transmission electron microscopy (HR-TEM) analysis (JEM 2100 Plus, JOEL, Japan) of optimized formulations was done at 80 kV. A drop of the diluted sample was placed on the carbon-coated copper grid and dried properly. The extra liquid was removed using tissue paper. The sample was stained and dried. The prepared samples were analyzed at high resolution. The surface roughness of the optimized transferosomes was analyzed using atomic force microscopy (AFM) (NANOSURF NG, Liestal, Switzerland). For AFM analysis, a thin film was obtained by placing a drop of dispersion on a thin glass slide using a spin coater. Images were captured at 300 kHz cantilever frequency, 256×256-pixel resolution, and a nominal force constant of 48 N m^-1^ at room temperature [22].

#### Determination of deformability

The deformability of the optimized formulation (formulation DDT-10) was determined by the extrusion method. Briefly, the vesicle size of the optimized formulation was determined by DLS before passing them through the extruder. The vesicle size was further determined after passing through the extruder containing a polycarbonate membrane filter of pore size 100 nm. The deformability of the transferosomes was calculated using Equation (4) [23]:


(4)

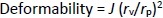



where *D* is the deformability index, *J* is the amount of transferosome suspension extruded, *r*_v_ is the vesicle size after extrusion, *r*_p_ is the pore size of the membrane.

#### Antioxidant assay of transferosomes

The antioxidant activity was determined by the DPPH (2,2-diphenyl-1-picrylhydrazyl) assay method. The DPPH agent used for free radical formation exhibited absorption band at 517 nm. Eight different concentrations (3.9, 7.8, 15.6, 31.25, 62.5, 125,250, and 500 μg/mL) of ascorbic acid (standard sample) and optimized transferosomes (test sample) were prepared and mixed with 0.004 % w/v solution of DPPH. The mixture was incubated for 15 minutes under dark conditions at 25 °C. The solution color changed from purple to yellow after incubation. The sample absorbance was recorded at 517 nm using a UV spectrophotometer (UV-1900, Shimadzu, Japan). The antioxidant activity was determined using Equation 5. The IC_50_ (half-maximal inhibitory concentration) value of optimized transferosomes was determined and compared against ascorbic acid (antioxidant standard) [24].


(5)

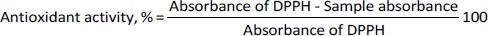



#### *In vitro* release studies

The formulated transferosomes were subjected to release studies using a USP type II dissolution apparatus. The dialysis membrane was activated by heating it in a sodium bicarbonate solution for 15 min, followed by washing it with double-distilled water. Transferosomes (equivalent to 5 mg of berberine HCl and 5 mg of diacerein) were taken into the dialysis membrane and placed into the dissolution apparatus (filled with PBS pH 7.4). The temperature of the dissolution medium was maintained at 37±0.5 °C. The paddles were rotated at 100 rpm. The samples (5 mL) were withdrawn in triplicate at different intervals (0, 1, 2, 3, 4, 5, 6, 8, 10, 12, 16 and 24 h). The sink condition was maintained by adding 5 mL of PBS (pH 7.4) immediately after each sampling. The samples were filtered and analyzed at 340 nm for berberine HCl and 258 nm for diacerein using a UV spectrophotometer (UV-1900, Shimadzu, Japan). The cumulative drug release was calculated and plotted against time [13].

Berberine HCl and diacerein release kinetics from dual delivery transferosomes were assessed for zero order (cumulative drug release against time) and first order (log of cumulative drug remaining against time) kinetics. Cumulative percent drug release against the square root of the time plot was used to confirm the drug release kinetics (Higuchi’s model). To confirm the drug diffusion mechanism (non-Fickian or Fickian), the *in vitro* release data was analyzed according to the Korsmeyer-Peppas model (log cumulative percent drug release against log time). The Hixson-Crowell model was applied to the *in vitro* drug release data (cube root of drug amount remaining in matrix versus time). The release mechanism assessment was based on the regression coefficient value [18]. PCP Disso V3 software (PCP, BVDU, Pune, India) was used to plot calibration curves and determine the cumulative drug release and kinetic modeling.

#### *Ex vivo* permeation studies

*Ex vivo* skin permeation studies were performed using hairless pig ear skin collected from a slaughterhouse (Dehradun, India). The skin was washed with PBS (pH 7.4), packed, and stored in a freezer at -8 °C. The study was carried out using a Franz diffusion cell in which pig skin was mounted between the donor and receiver compartments. The formulation was applied to the skin uniformly. The outer layer of the skin was set towards the facing donor compartment and the internal layer towards the receiver compartment, which was filled with PBS (pH 7.4). Samples (2 mL) were withdrawn at different time intervals (0, 1, 2, 3, 4, 5, and 6 h). The sink condition was maintained by adding 2 mL of fresh buffer solution immediately after each sampling. Sample analysis was done in triplicate at 340 and 258 nm for berberine HCl and diacerein, respectively. The cumulative drug permeation was calculated according to the skin surface area against time to estimate the permeability coefficient and flux *J*_ss_ (μg cm^-2^ h^-1^) [21].

#### Raman analysis

For *in vitro* permeation studies, optimized formulation, pig skin, and pig skin containing optimized formulation samples were analyzed to see how well the formulation could pass through the skin using Direct coupled universal modular Raman spectrometer with Imaging (RIMS-U-DC, Rinztech Nz Ltd., New Zealand). The pig skin was mounted on the glass slide using forceps, ensuring stratum corneum facing towards the lens. The sample was observed at 20× at 785 nm and a laser power of 300 mW, followed by laser stability of 1 %. In addition, all the other pig skin samples were analyzed under the same conditions, and data were obtained, analyzed, and reported [25].

#### Skin irritation test

A skin safety study was carried out using shaved and excised dorsal skin (2 cm^2^) of male BALB/c mice (10 weeks old, 20 to 22 g). The study protocol was approved by the Institutional Animal Ethics Committee of Central Animal House Facility UPES University, Dehradun, India (UPES/IAEC/2023/3/07 dated 20/12/2023). Psoriasis was induced by applying 62.5 mg day^-1^ of imiquimod cream (Imiquad® cream, Glenmark Pharmaceuticals, Mumbai, India) to the skin for 6 consecutive days. Optimized transferosomes (formulation DDT-10) and normal saline as reference were applied separately onto the depilated dorsal skin of mice from day 3 to day 6 [26]. The skin was observed for any evident transformation on day 7 by visual scoring using the modified Draize method. The erythema scores were graded from 0 to 4 as follows: 0 with no sign of redness; 1 with trial erythema (hardly seeming light pink), 2 with moderate erythema (dusky pink), 3 with moderate to high erythema (bright red), and 4 high levels of erythema (dark red) [27].

#### Stability studies

The optimized formulation was subjected to stability assessment at different storage conditions, such as 4±1 °C and 25±2 °C / 60±5 % of relative humidity (RH) for 3 months was carried out [28]. The results of the stability study were reported in terms of physical appearance, vesicle size, morphology, and zeta potential.

## Results and discussion

### Optimization studies

The dual delivery loaded transferosomes were prepared using the thin film hydration method and optimized using the Box-Behnken design. Three independent variables, *A*, *B* and *C*, were used to build a significant model in the Box- Behnken design. The interactions between individual variables and their effects on experimental responses *i.e*., vesicle size and entrapment efficiency, were optimized using a 3-level factorial design (Tables 1 and 2). The model's significance was determined based on statistical parameters comprising the ANOVA and regression coefficient (*R*^2^) test. Formulation DDT-10 (85 : 15 ratio of phosphatidylcholine (*A*) and sodium deoxycholate (*B*) at 3 min sonication time (*C*) was found as an optimized formulation (Tables 1 and 2).

**Table 2.** Results of berberine HCl and diacerein loaded dual delivery transferosomes.

Formulation code	Particle size, nm	Entrapment efficiency, %		Drug loading, %		Deformability, %
		Berberine HCl	Diacerein	Berberine HCl	Diacerein	
DDT-1	95.93 ±2.5	69.14±1.4	71.32±2.2	12.94±0.03	15.03±0.65	8.47±0.02
DDT-2	118.45±1.4	65.30±1.7	72.34±1.4	12.70±0.24	15.09±0.39	13.11±0.09
DDT-3	103.00±1.2	62.11±1.4	66.61±1.5	12.35±0.11	14.59±0.02	10.79±0.19
DDT-4	114.60±3.5	59.24±1.5	64.87±1.6	12.04±0.02	14.47±0.20	12.97±0.02
DDT-5	104.50±2.2	85.73±1.5	88.22±1.8	17.05±0.07	19.37±0.36	11.47±0.36
DDT-6	90.80±2.7	68.32±2.4	65.51±1.2	12.90±0.07	14.51±0.14	8.35±0.18
DDT-7	99.90±1.6	64.51±1.6	69.11±2.1	12.50±0.04	15.07±0.05	9.78±0.12
DDT-8	119.00±2.8	72.11±2.1	84.09±1.4	15.7±0.19	18.96±0.16	14.51±0.12
DDT-9	106.90±2.6	60.27±1.4	61.15±1.4	12.16±0.09	14.40±0.07	10.80±0.11
DDT-10	110.90±2.8	89.50±1.5	91.23±1.8	19.41±0.47	21.91±0.05	12.44±0.39
DDT-11	115.00±1.6	75.42±1.2	74.42±1.5	16.17±0.06	15.90±0.65	13.34±0.25
DDT-12	86.98±2.4	66.16±2.4	68.61±2.3	12.77±0.02	14.98±0.28	7.43±0.04
DDT-13	121.45±2.5	76.00±1.2	82.32±1.5	16.70±0.21	18.22±0.07	14.15±0.08

**Figure 1.**
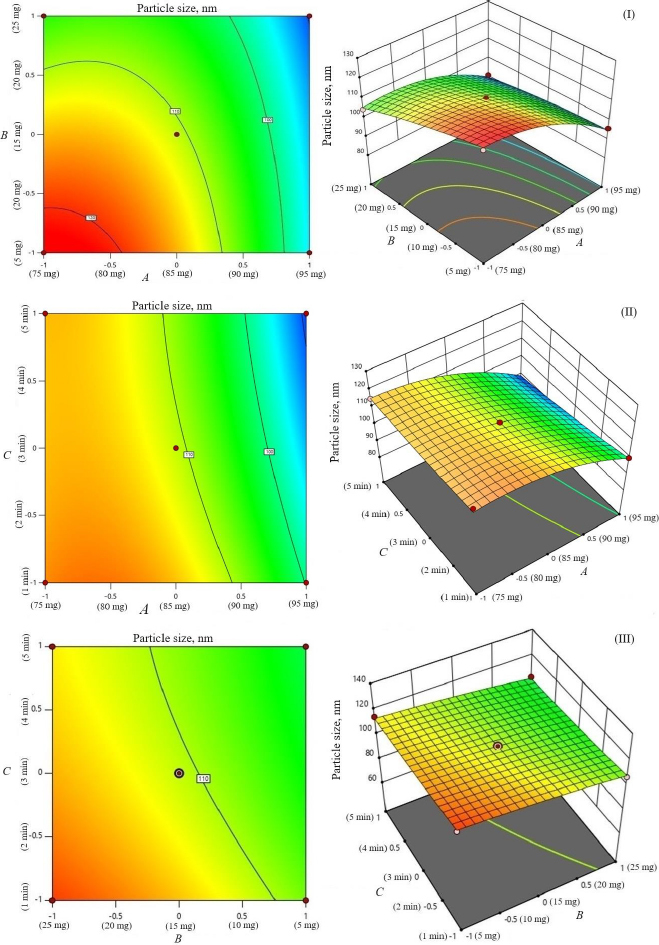
Contour plots and 3D response surface of parameters as a function of formulation variables (particle size): amount of phosphatidylcholine and amount of sodium deoxycholate (I), sonication cycles and amount of phosphatidylcholine (II), and sonication time and amount of sodium deoxycholate (II).


The study investigated the impact of independent variables on the particle size and entrapment efficiency of dual-loaded drugs (Table 2). Contour plots and 3D response plots are shown in Figure 1. The quadratic equations were developed to represent the relationship between the independent and dependent variables (Eq. 6-8).

(6)

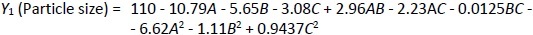




(7)

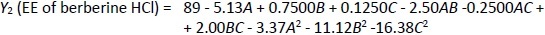




(8)

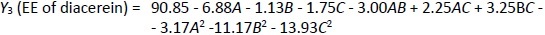



where *A* is the amount of phospholipid, *B* is the amount of edge activator, *C* is the sonication cycle time, *AB* is the amount of the phospholipid and edge activator, *AC* is the amount of phospholipid and sonication cycle time, *BC* is the amount of edge activator and sonication cycle time.

The negative magnitude of the amount of phospholipid reduces the particle size of transferosomes (Equation (6)). Previous studies have also documented a reduction in vesicle size at elevated amounts of phospholipid [29,30]. The amount of edge activator (sodium deoxycholate) also had a negative effect on vesicle size (Equation (6)). Therefore, a higher level of phospholipid and edge activator (sodium deoxycholate) is suggested for producing transferosomes of smaller particle size because of the higher flexibility of the lipid bilayer. The relationship between the dependent and independent variables was further elucidated using response surface plots. Figure 1 shows that the mean vesicle size decreases with an increase in phospholipid amount. Sonication cycle time has a suppressive effect on the vesicle size, as a higher level of sonication cycle decreases the aggregation of particles and reduces the vesicle size.

**Figure 2.**
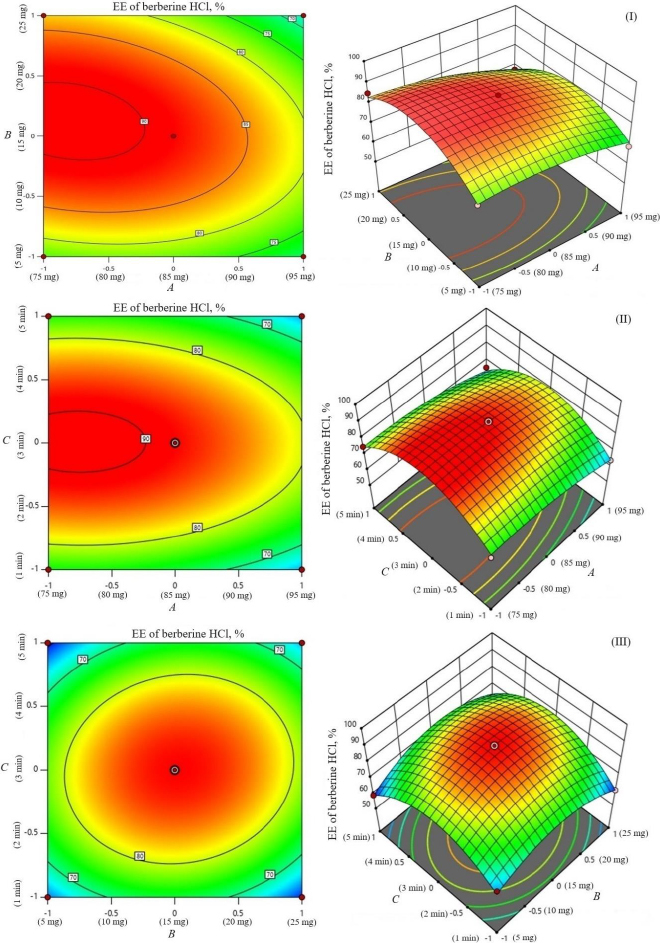
Contour plots and 3D response surface of parameters as a function of formulation variables (entrapment efficiency of berberine HCl): amount of phosphatidylcholine and amount of sodium deoxycholate (I), sonication time and amount of phosphatidylcholine (II), and sonication cycles and amount of sodium deoxycholate (III).

**Figure 3.**
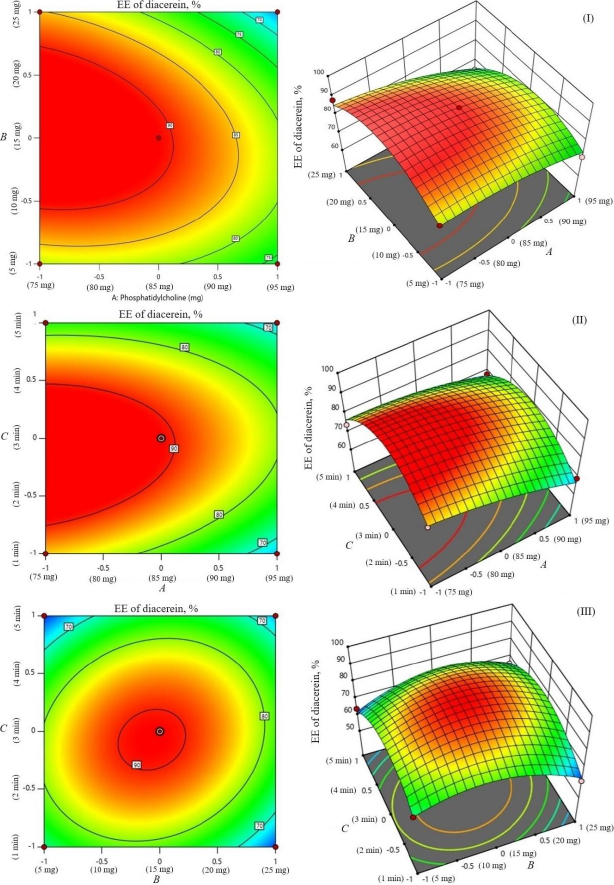
Contour plots and 3D response surface of parameters as a function of formulation variables (entrapment efficiency of diacerein): amount of phosphatidylcholine and amount of sodium deoxycholate (I), sonication time and amount of phosphatidylcholine (II), and sonication time and amount of sodium deoxycholate (III).

**Figure 4.**
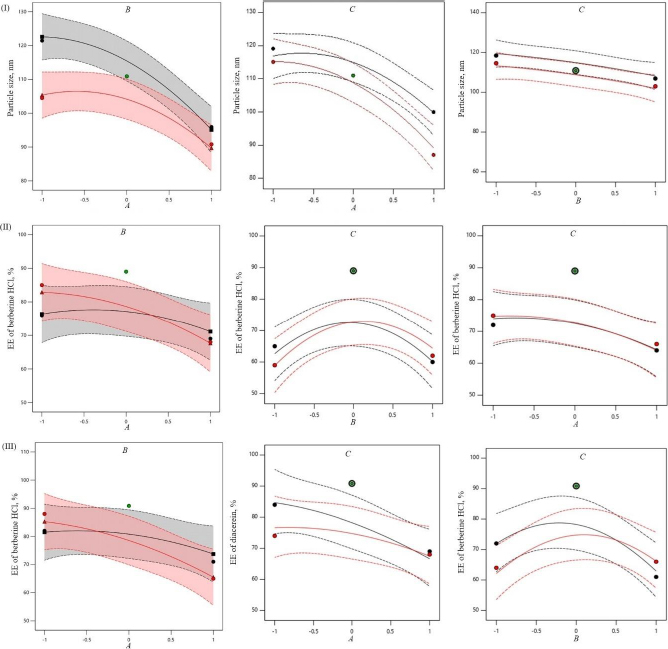
Interaction plots showing probable interaction between the amount of phosphatidylcholine (phospholipid) and amount of sodium deoxycholate (edge activator), sonication time and amount of phosphatidylcholine, and sonication time and amount of sodium deoxycholate affecting particle size (I), entrapment efficiency of berberine HCl (II), and entrapment efficiency of diacerein (III).

The encapsulation of berberine HCl and diacerein was high (59.24±1.5 to 89.50±1.5 % and 61.15±1.4 to 91.23±1.8 %, respectively) in all the formulations with good loading efficiency (12.04±0.02 to 19.41±0.47 % and 14.40±0.07 to 21.91±0.05 %, respectively), which might be due to the poor aqueous solubility of the drugs. Phosphatidylcholine had a negative correlation with the encapsulation of berberine HCl and diacerein (Equations (7) and (8)). This means that higher levels of phosphatidylcholine decrease the drug encapsulation in the transferosomes [31]. So, a lower level of phosphatidylcholine is suggested for high drug encapsulation. On the other hand, sodium deoxycholate had a positive correlation with berberine HCl encapsulation (Equation (7), Figure 2), and a negative correlation with diacerein encapsulation (Equation (8), Figure 3). However, a negative effect of edge activator concentration on drug entrapment efficiency has been reported earlier [32]. The presence of a higher surfactant content may lead to a decrease in entrapment efficiency [33,34]. This could be attributed to the elevated permeability of the vesicle membrane caused by the organization of surfactant molecules within the lipid bilayer structure of the vesicle. This arrangement may create openings in the vesicle membrane, resulting in enhanced fluidity and causing the drug contained within to leak out [10,35]. A higher level of surfactant can increase the number of vesicles produced, resulting in a greater volume of the hydrophobic bilayer region that can accommodate hydrophobic drugs [10]. This could be a possible reason for the high entrapment of diacerein. Table 3 shows that there was a good degree of agreement between the observed and predicted values, indicating the validity of the established model. The probable interactions between phosphatidylcholine (phospholipid), sodium deoxycholate (edge activator), and affecting particle size (*A*), and entrapment efficiency of berberine HCl (*B*) and diacerein (*C*) were systematically investigated (Figure 4). This study showed a higher drug encapsulation and loading, surpassing the findings of a previously reported study [20].

**Table 3.** Actual and predicted values for all response variables for different batches.

Formulation code	Mean particle size, nm		Encapsulation efficiency, %			
			Berberine HCl		Diacerein	
	Actual	Predicted	Actual	Predicted	Actual	Predicted
DDT-1	95.93	95.08	69.00	71.12	71.00	73.75
DDT-2	118.45	119.46	65.00	62.62	72.00	71.87
DDT-3	103.00	101.98	62.00	64.37	66.00	66.12
DDT-4	114.60	113.32	59.00	58.87	64.00	61.87
DDT-5	104.50	105.35	85.00	82.87	88.00	85.25
DDT-6	90.80	89.67	68.00	67.62	65.00	65.50
DDT-7	99.90	99.74	64.00	64.25	69.00	66.37
DDT-8	119.00	116.86	72.00	74.00	84.00	84.62
DDT-9	106.90	108.18	60.00	60.12	61.00	63.12
DDT-10	110.90	110.9	89.00	89.00	90.85	90.85
DDT-11	115.00	115.15	75.00	74.75	74.00	76.62
DDT-12	86.98	89.11	66.00	64.00	68.00	67.37
DDT-13	121.45	122.57	76.00	76.37	82.00	81.50

The amount of phosphatidylcholine (*A*) had a relatively greater influence (*p* < 0.05) on vesicle size (*Y*_1_) (*F* value 151.47) than the amount of sodium deoxycholate (*B*) (*F* value 41.57) and sonication cycle time (*C*) (*F* value 12.37). Sonication cycle time showed a suppressive effect on vesicle size. The combination of phosphatidylcholine and sodium deoxycholate (*AB*) significantly influenced the vesicle size (*F* value 5.68 and *p* value 0.0974). In comparison to the effect of the combination of variables, the influence squared form of phosphatidylcholine amount (*A*^2^) had a higher effect on vesicle size (*F* value 16.30) (Table 4).

**Table 4.** Results of One Way ANOVA for particle size and entrapment efficiency of berberine HCl and diacerein in transferosomes optimized by Box Behnken design.

Response	Source	Sum of squares	Mean square	*F*-value	*p*-value
Y_1_ Particle size (reduced quadratic model)	Model	1459.88	162.21	26.37	0.0105*
	*A*	931.82	931.82	151.47	0.0012
	*B*	255.72	255.72	41.57	0.0076
	*C*	76.08	76.08	12.37	0.0390
	*AB*	34.93	34.93	5.68	0.0974
	*AC*	19.89	19.89	3.23	0.1700
	*BC*	0.0006	0.0006	0.0001	0.9926
	*A* ^2^	100.28	100.28	16.30	0.0273
	*B* ^2^	2.80	2.80	0.4547	0.5484
	*C* ^2^	2.04	2.04	0.3309	0.6054
	Residual	18.46	6.15		
	Cor total	1478.34			
Y_2_ Entrapment efficiency of berberine HCl	Model	989.25	109.92	11.47	0.0347*
	*A*	210.13	210.13	21.93	0.0184
	*B*	4.50	4.50	0.4696	0.5424
	*C*	0.1250	0.1250	0.0130	0.9163
	*AB*	25.00	25.00	2.61	0.2047
	*AC*	0.2500	0.2500	0.0261	0.8820
	*BC*	16.00	16.00	1.67	0.2868
	*A* ^2^	26.04	26.04	2.72	0.1979
	*B* ^2^	282.89	282.89	29.52	0.0122
	*C* ^2^	612.89	612.89	63.95	0.0041
	Residual	28.75	9.58		
	Cor total	1018.00			
*Y*_3_ Entrapment efficiency of diacerein	Model	1088.74	120.97	9.25	0.0468*
	*A*	378.13	378.13	28.90	0.0126
	*B*	10.13	10.13	0.7739	0.4438
	*C*	24.50	24.50	1.87	0.2646
	*AB*	36.00	36.00	2.75	0.1957
	*AC*	20.25	20.25	1.55	0.3018
	*BC*	42.25	42.25	3.23	0.1702
	*A* ^2^	23.04	23.04	1.76	0.2764
	*B* ^2^	285.44	285.44	21.82	0.0185
	*C* ^2^	443.21	443.21	33.88	0.0101
	Residual	39.25	13.08		
	Cor total	1127.99			

*significant values

The amount of phosphatidylcholine, amount of sodium deoxycholate and sonication cycle time exhibit significant effects on entrapment efficiency (*Y*_2_ and *Y*_3_) at *p* < 0.05. The amount of phosphatidylcholine (*A*) had a relatively greater influence on entrapment efficiency (Y_2_ and Y_3_) than the amount of sodium deoxycholate (*B*) and sonication cycle time (*C*) as indicated by measured *F* values (21.93 and 28.90, respectively for berberine HCl and diacerein).

Sonication time showed a suppressive effect on entrapment efficiency. The combination of phosphatidylcholine and sodium deoxycholate (*AB*) significantly influenced the entrapment of berberine HCl (*F* value 2.61 and *p* value 0.2047). While the combination of sodium deoxycholate and sonication time (*BC*) significantly influenced the entrapment of diacerein (*F* value 3.23 and *p* value 0.1702). No interaction was observed between the phosphatidylcholine amount and sonication time (*AC*) on the entrapment of berberine HCl. In comparison to the effect of the combination of variables, the influence squared form of sodium deoxycholate amount (*B*^2^) and sonication time (*C*^2^) had a higher effect on the entrapment of berberine HCl (*F* value 29.52 and 63.95, respectively) and diacerein (*F* value 21.82 and 33.88, respectively). The model reliability and precision were confirmed using the coefficient of variation (Table 4).

### Vesicle size, polydispersity index, and zeta potential

**Figure 5.**
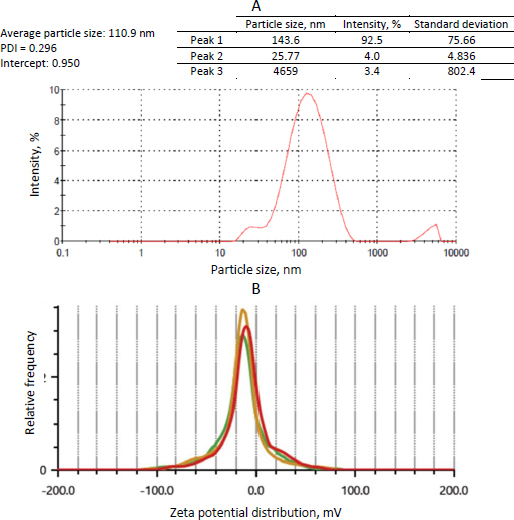
Results of A - particle size distribution and PDI and B – average zeta potential analysis of optimized formulation (formulation DDT-10) (test 1: red, test 2: yellow, test 3: green).

The particle size of the transferosomes ranged from 86.98±2.4 to 121.45±9.22 nm, while the PDI ranged from 0.244±0.014 to 0.452±0.008. The optimized formulation (formulation DDT-10) had a particle size of 110.9±2.8 nm, a PDI of 0.296 (Figure 5A), and a ζ potential of -13.3±0.6 mV (Figure 5B). The particle size is important for cellular absorption and colloidal stability, and the size distribution is controlled by PDI. The endocytosis process is primarily responsible for nanoparticle cellular uptake. In the case of transdermal distribution, a particle size of less than 300 nm is easily deposited into the epidermis and dermis layers of the skin and may even reach the sub-dermis layer. Particles within the size range of 10-210 nm can penetrate through a trans-follicular pathway, while particles smaller than 7 nm can penetrate by lipidic trans-epidermal channels. A PDI value exceeding 0.7 indicates that the system exhibits an undesirable wider range of particle sizes. PDI values for vesicular nanoparticles are less than 0.3, suggesting a homogenous system and reduced aggregation [13,36,37].

Transferosomes acquire their flexibility and deformability characteristics through the destabilization of the lipid layers induced by the edge activator. The reduction in particle size observed when sodium deoxycholate interacts with lipid layers can be attributed to its negative charge. The stability of a nanoparticle system can be assured by measuring its zeta potential, which offers information on surface charge and electrostatic repulsion induced by repulsive energy between particles. When the Van der Waals forces within the system become dominant and the electrostatic repulsion increases, the system is deemed to be in a stable state. ζ potential is influenced by particle distribution, accumulation, and flocculation. Vesicular structures containing anionic surfactants often exhibit less aggregation due to the presence of electrostatic repulsion. The ζ potential of transferosomes is steady between +30 and -30 mV [13,38]. Based on the outcomes of our investigation, it was observed that the optimized formulation (formulation DDT-10) displayed a uniform size distribution, favorable stability, and absence of aggregation, indicating its potential for skin permeation.

### Fourier-transformed infrared analysis

**Figure 6.**
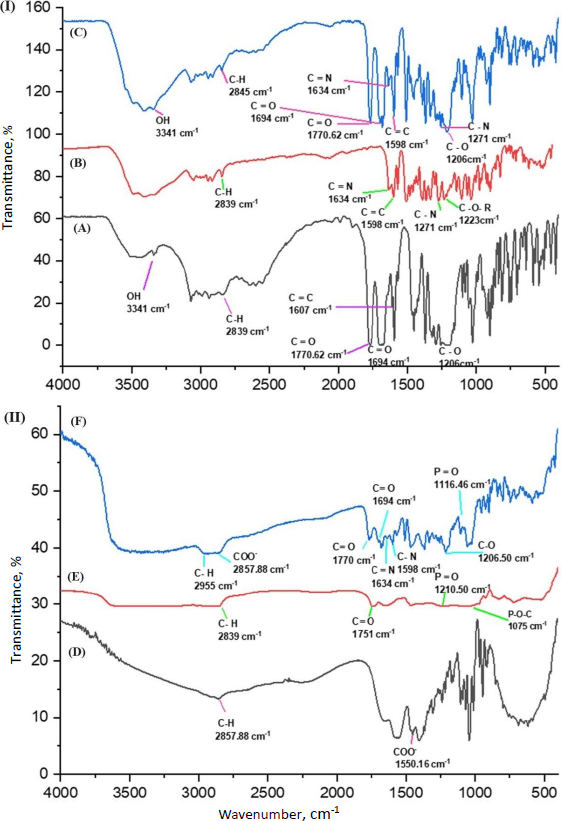
FTIR spectrum of: A - diacerein, B - berberine HCl, C - a physical mixture of diacerein and berberine HCl, D - sodium deoxycholate, E - phosphatidylcholine and F - optimized formulation (formulation DDT-10).

Berberine HCl, diacerein, their physical mixture, phosphatidylcholine, and sodium deoxycholate were subjected to FTIR analysis to determine the presence of the functional groups and their interactions, if any (Figure 6). The characteristic peaks of berberine HCl, diacerein, a physical mixture of berberine HCl and diacerein, phosphatidylcholine, and sodium deoxycholate are presented in Table 5. No new peaks were identified, and both drugs were found to be compatible with each other.

**Table 5.** Functional groups present in berberine HCl, diacerein, physical mixture of diacerein and berberine HCl, sodium deoxycholate, phosphatidylcholine, and optimized formulation (formulation DDT-10) and their quantified frequencies.

Sample	Wavenumber, cm^-1^	Remark	Ref.
Berberine HCl	2839	C-H stretching	[39-42]
	1634	Iminium C=N^+^ double bond	
	1598	C=C stretching	
	1271	C-O-C stretching	
	1223	C-O stretching	
Diacerein	3341	O-H stretching band of -COOH	[42-45]
	2839	C-H stretching of an aliphatic symmetric group	
	1770	C=O stretching band of an ester group	
	1694	C=O stretching band of -COOH	
	1607	C=O stretching of an aromatic group	
	1206	C-O stretching	
Physical mixture of berberine HCl and diacerein	3341	O-H stretching	
	2845	C-H stretching	
	1770	C=O stretching band of an ester group	
	1694	C=O stretching band of -COOH	
	1634	Iminium C=N+ double bond	
	1598	C=C stretching	
	1271	C-O-C stretching	
	1206	C-O stretching	
Phosphatidylcholine	2839	C-H stretching	[46,47]
	1751	C=O stretching	
	1210	P=O stretching	
	1075	P-O-C stretching	
Sodium deoxycholate	2857	C-H stretching	[48,49]
	1550	COO^-^ stretching	
Formulation DDT-10	2995	C-H stretching	
	1770	C=O stretching band of an ester group	
	1694	C=O stretching band of -COOH	
	1634	Iminium C=N+ double bond	
	1598	C=C stretching	
	1206	C-O stretching	
	1116	P=O stretching	

### X-ray diffraction analysis

**Figure 7.**
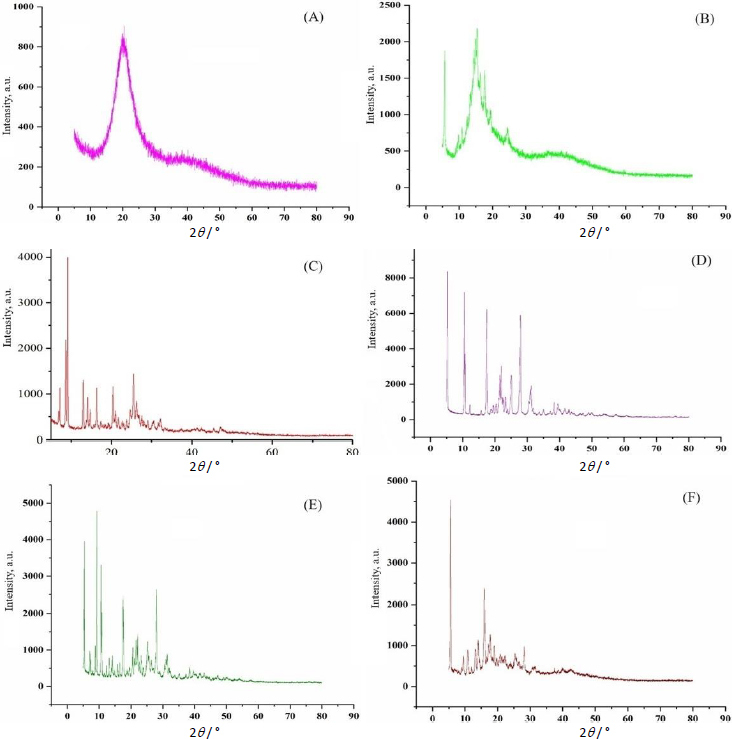
X-ray diffraction spectrum of A - phosphatidylcholine, B - sodium deoxycholate, C - berberine HCl, D - diacerein, E - physical mixture of berberine HCl and diacerein and F - optimized formulation (formulation DDT-10).

X-ray diffraction (XRD) analysis of berberine HCl, diacerein, a physical mixture of berberine HCl and diacerein, and optimized formulation (formulation DDT-10) was performed to assess the physical form of drugs in their pure form, physical mixture, and optimized formulation. The diffraction angles and sharp peaks of berberine HCl were found at 6.8, 7.5, 8.6, 12.9, 16.3, 20.4 and 25.5° [39,40]. Diacerein showed sharp peaks at 5.2, 10.5, 17.4, 21.9, 25.1 and 27.8°, exhibiting its crystallinity. A similar diffraction pattern of diacerein has been reported earlier [50]. Peaks at 5.4, 10.3, 12.4, 17.39 and 27.25° in the diffractogram of the berberine HCl and diacerein physical mixture indicate no change in the physical form of the mixture. A major broad peak of phosphatidylcholine and a peak of sodium deoxycholate were recorded at 21.02 and 15.43°, respectively. The optimized formulation (DDT-10) exhibited peaks at 5.56, 10.88, 13.99, 15.92, 17.7 and 28.23° with reduced intensity, indicating the partial conversion of drug molecules into the amorphous phase (Figure 7).

### Thermogravimetric analysis

**Figure 8.**
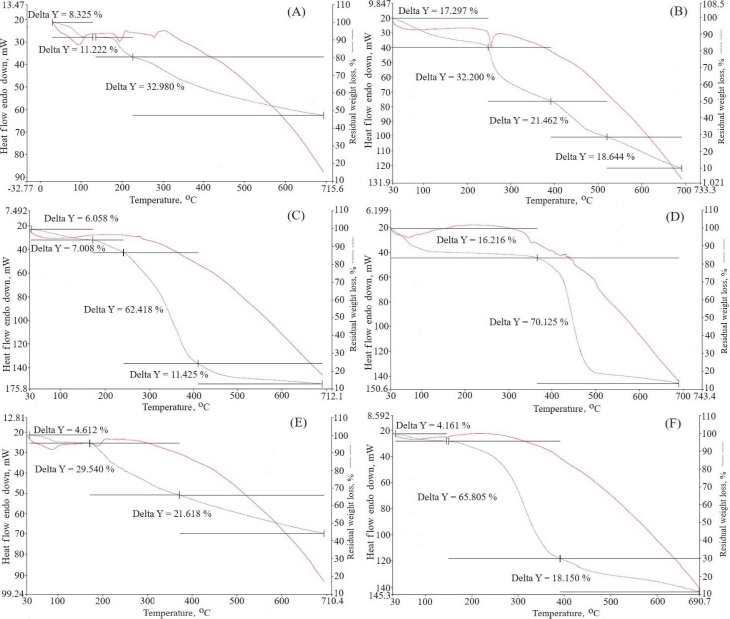
Results of thermogravimetric analyses of A - berberine HCl, B - diacerein, C - phosphatidylcholine, D - sodium deoxycholate, E - physical mixture of diacerein and berberine HCl and F - optimized formulation (formulation DDT-10). The curve representing Delta Y indicates residual weight loss.

Thermogravimetric analysis was used to ascertain the temperature dependence of weight loss (Figure 8). Percentage weight loss is represented as delta Y. Pure berberine HCl exhibited three stages of weight loss: near 100, 180 and above 280 °C. This weight loss occurred due to the decomposition of the water [51]. Pure diacerein exhibited weight loss above 250 °C due to water decomposition. The physical mixtures of berberine HCl and diacerein showed no temperature change and found weight loss near 100 and above 200 °C. The major reason for weight loss is due to the slow elimination of the methoxy group [52]. The phosphatidylcholine did not depict any peak. Sodium deoxycholate exhibited a peak near 350 °C. Formulation DDT-10 exhibited a peak near 150 °C, which signifies weight loss due to the entrapment of the drug molecules in the lipid bilayers and water loss [20].

### Morphological characterization

**Figure 9.**
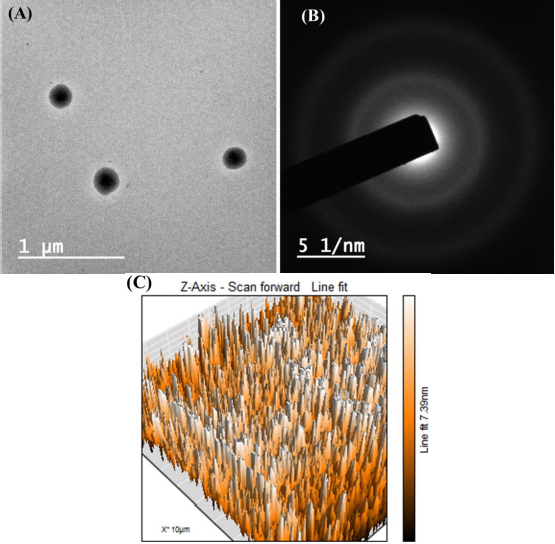
Results of berberine and diacerein-loaded optimized transferosomes (formulation DDT-10): A - TEM image, B - Selected area electron diffraction (SAED) image and C - 3D AFM image.

TEM images revealed the spherical shape of the transferosomes (formulation DDT-10) without aggregation of particles (Figure 9A). The vesicle size in TEM analysis was found to be near the DLS result and can be acceptable for topical administration. However, there is no fundamental linkage between the TEM and DLS results because DLS works on the Brownian motion principle, and in TEM, image formation occurs due to the passing of the electron flux through the sample. Moreover, TEM is a number-based technique, while DLS is an intensity-based technique. In DLS, particles show larger diameters because of the development of hydrating layers around the particles, and it calculates hydrodynamic diameter, while TEM discloses the projected surface area of particles.

Selected area electron diffraction (SAED) analysis reveals the crystallinity of loaded molecules. The appearance of the diffused rings signifies the loaded crystallinity of the molecule. The optimized formulation (formulation DDT-10) did not show any diffused layers in the SAED pattern, which confirmed that drug molecules are not in crystalline form (at least not at a larger scale) (Figure 9B). Surface roughness has a great impact on cellular uptake. AFM analysis is used to analyse surface topography information from 2D and 3D views [18,53]. The optimized formulation (formulation DDT-10) had Ra (average roughness) values of 5.43 nm and *R*_q_ (root mean square) values of 6.58 nm (Figure 9C).

### Deformability

Edge activator is responsible for transferosome deformability, which permits them to infiltrate the stratum corneum due to their squeezing and deforming characteristics. The charge on the edge activator influences transferosome characteristics. The results showed that the deformability of the prepared transferosomes ranged between 7.43±0.04 to 14.51±0.12 % (Table 2). The deformability of optimized formulation (formulation DDT-10) was 12.44±0.39 %. This signifies that the amount of sodium deoxycholate has an effective impression on the lipid bilayers, serves flexibility in transferosomal structure without any rupturing, and provides the potential to squeeze when applied topically [54].

### Antioxidant activity

Oxidative stress occurs when there is an imbalance between the levels of oxidants and antioxidants in the body, which in turn contributes to the formation of reactive oxygen species (ROS). Oxidative stress, caused by ROS, has a notable impact on apoptosis, autophagy, and regeneration processes and contributes to the development of psoriasis [55]. Both berberine HCl and diacerein possess the ability to remove ROS and demonstrate antioxidant properties [56,57]. It has been reported that these molecules can suppress the activity ROS in a concentration-dependent manner. Due to their high phenolic group content, berberine and diacerein can donate electrons and improve ROS scavenging [58]. Berberine HCl can increase the levels of endogenous antioxidants like catalase, superoxide dismutase, and glutathione peroxidase. It also promotes the downregulation of oxidative stress markers such as glutathione and malondialdehyde [59]. The current investigation involved the utilization of ascorbic acid as a reference compound, which demonstrated an *IC*_50_ value of 14.2±0.1 μg mL^-1^. Additionally, the optimized formulation exhibited an *IC*_50_ value of 36.42 μg mL^-1^.

### In vitro release study and release kinetics

**Figure 10.**
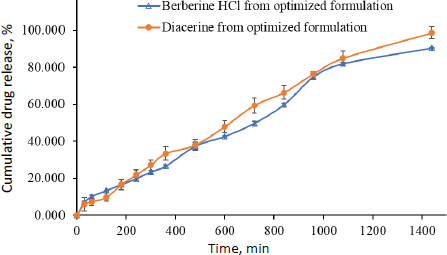
Dissolution profile of berberine HCl (empty triangles) and diacerein (filled circles) from the optimized transferosomes (formulation DDT-10) in PBS pH 7.4 (data presents mean±SD, *n* = 3).

A study was performed to examine the extended-release of berberine HCl and diacerein from the optimized transferosomes in alkaline media (pH 7.4). The study aimed to get a better understanding of the mechanism of drug release. The *in vitro* dissolution study was conducted for 24 h utilizing a dialysis bag method. After 18 h, the release of berberine HCl and diacerein from the optimized formulation was found to be 82.09±0.81 and 85.02±3.81 %, respectively. The results demonstrated that the optimized transferosomes exhibited a sustained release of berberine HCl and diacerein, potentially enhancing the therapeutic efficacy of both drugs. The dissolution study was terminated after 24 h when 90.546±0.21 % of berberine HCl and 98.730±3.21 % of diacerein were released (Figure 10).

The most appropriate kinetic model was selected based on its goodness of fit, determined by the highest *r*^2^ value. In the present study, the release characteristics of berberine HCl and diacerein from the optimized transferosomes were best explained by zero-order kinetics with *r*^2^ values of 0.9850 and 0.9901, respectively. The linearity and correlation coefficient values of Higuchi's plot suggest that the release profiles are regulated by diffusion. Peppas’ model showed good linearity for berberine HCl (*r*^2^ = 0.9834) and diacerein (*r*^2^ = 0.9893) from the optimized transferosomes in alkaline media (pH 7.4). According to Peppas' theory, Fickian diffusion controls the drug's release when the value of *n* is 0.43. When the value of n is between 0.43 and 0.85, the drug release follows anomalous (non-Fickian) diffusion. When the value of n is 0.85, the drug release is characterized by case II transport, which implies the expansion of the polymer matrix. Finally, when the value of *n* is greater than 0.85, the drug release is described as super-case II transport. In the present study, best fitting was obtained with n values of 0.43 < *n* < 0.85 for berberine HCl (*n* = 0.6987) and diacerein (*n* = 0.8010), which indicates anomalous (non-Fickian) diffusion of loaded drugs from the developed transferosomes (Table 6).

**Table 6:** Results of kinetic modeling of berberine HCl and diacerein release data from optimized transferosomes (formulation DDT-10) in alkaline media (pH 7.4).

Kinetic model	Berberine HCl		Diacerein	
	*Coefficient of correlation (r^2^)*	*Rate constant (k)*	*Coefficient of correlation (r^2^)*	*Rate constant (k)*
Zero order	0.9850	0.0710	0.9901	0.0767
T-test	20.606	(Passes)	25.378	(Passes)
First order	0.9591	-0.0014	0.8831	-0.0019
T-test	12.224	(Passes)	6.786	(Passes)
Higuchi’s model	0.9407	2.0391	0.9461	2.2013
T-test	10.002	(Passes)	10.530	(Passes)
Korsmeyer-Peppas’ model*	0.9834	0.5171	0.9893	0.2890
T-test	19.559	(Passes)	24.400	(Passes)
Hixson Crowell model	0.9825	-0.0004	0.9688	-0.0004
T-test	19.034	(Passes)	14.094	(Passes)

*n value of berberine HCl and diacerein are 0.6987 and 0.8010, respectively

### Ex vivo permeation studies

**Figure 11.**
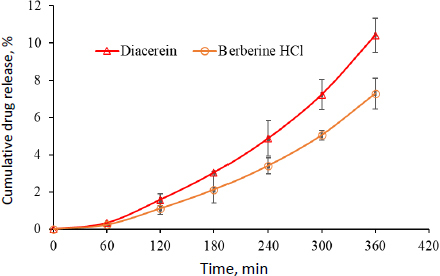
Results of *ex vivo* permeation studies in pig skin (data presents mean±SD, *n* = 3).

The *ex-vivo* permeation studies were performed using pig ear skin in the Franz diffusion assembly for 6 h. Pig skin has been shown to be a good surrogate for human skin and is commonly used in topical drug delivery for *in vitro* studies [60]. The cumulative drug permeation of berberine HCl and diacerein was found to be 7.286 and 10.41 μg cm^-2^, respectively. Berberine HCl exhibited a flux of 0.0224 μg cm^-2^ h^-1^ and a permeation coefficient (*K*_p_) of 2.24 cm h^-1^ and diacerein exhibited a flux of 0.0462 μg cm^-2^ h^-1^ and *K*_p_ of 4.62 cm h^-1^ (Figure 11). The results confirmed the movement of transferosomes through the skin via deformability property and the driving force of the osmotic gradient [61]. In addition, transferosomes are supposed to be passed through the stratum corneum, and drug partitioning occurs between the lipid part of both nanocarriers and the stratum corneum [13]. Amplification of stratum corneum membrane fluidity and intercellular lipids destabilization could be another possible reason for enhanced skin permeation [62].

### Raman analysis

**Figure 12.**
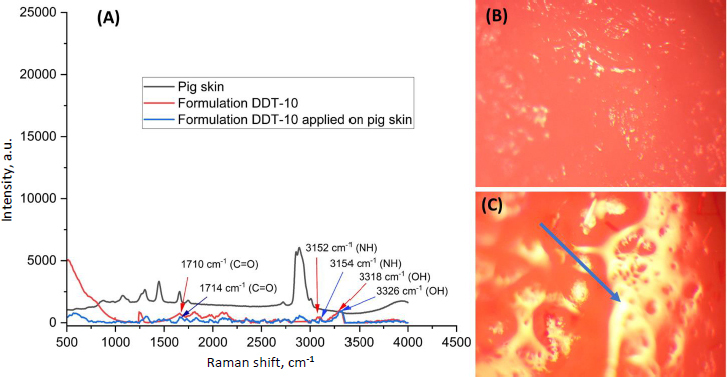
Results of Raman analysis: A - spectra of treated pig skin, B - image of blank pig skin and C - image of pig skin treated with optimized formulation (formulation DDT-10).

Raman spectra facilitated the identification of active vibrational modes in the target species. This was done by comparing the spectra obtained for blank pig skin, optimized formulation (formulation DDT-10), and pig skin treated with optimized formulation (formulation DDT-10). The presence of other chemicals can produce strong fluorescence signals that can disrupt and coincide with the Raman signal. This is particularly evident in intricate settings like the skin, where melanin absorbs visible light. So, the sample analysis was done at 785 nm (near-infrared reason). At this wavelength, the fluorescence intensity diminishes considerably, facilitating the accurate identification of Raman-active vibrational mode peaks [25]. In the present study, the optimized formulation (DDT-10) exhibited C=O at 1710 cm-1, broadened and shifted by H-bonding observed at 3152 cm^-1^ in the NH group and 3318 cm^-1^ in the OH group, respectively. In addition, the Raman spectra of the permeated optimized formulation in pig skin revealed the C=O functional group presence at 1714 cm^-1^, broadened and shifted by H-bonding observed at 3154 cm^-1^ in the NH group and at 3326 cm^-1^ in the OH group, respectively (Figure 12A). These spectral results confirmed the presence of berberine HCl and diacerein dual-loaded transferosomes that successfully permeated the stratum corneum because of their deformability property without any rupturing. The image of blank pig skin, observed in the Raman spectroscopy, exhibited a reddish part due to erythrocytes and other blood constituents (Figure 12B). On the other side, the transferosomes permeated pig skin exhibited a yellowish appearance that confirmed the permeation in the skin by transferosomes (Figure 12C). In addition, the D-band and G-band in the spectrum of optimized formulation and pig skin treated with optimized formulation (formulation DDT-10) appeared at 1322 and 1548 cm^-1^, respectively.

### Skin irritation test

**Figure 13.**
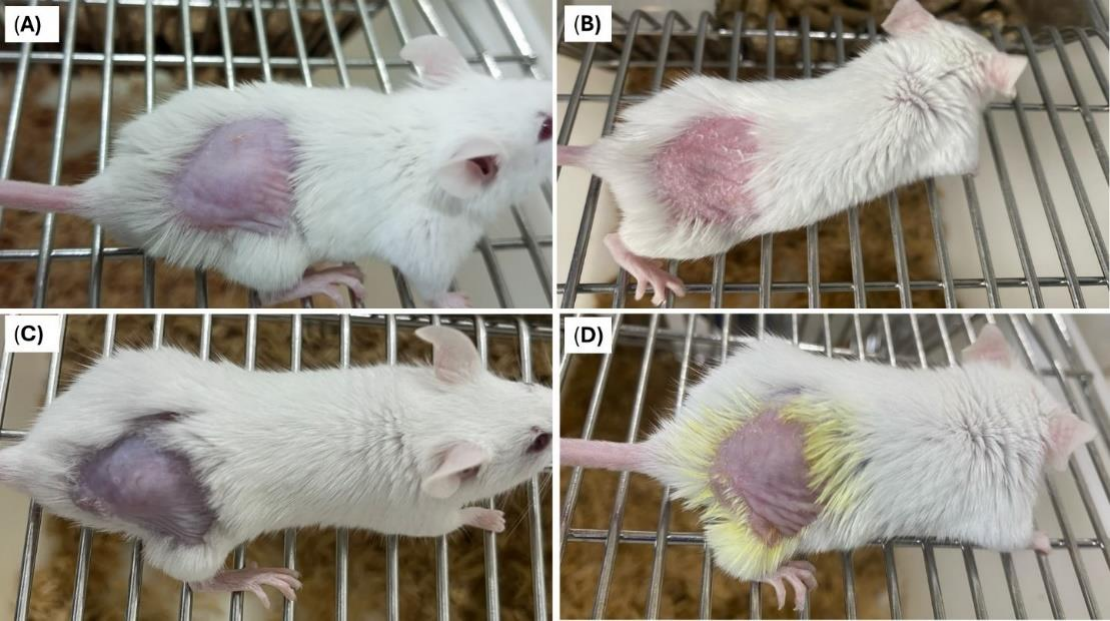
Visual assessment of skin irritation in Imiquad^®^ cream treated male BALB/c mice. Images A and C are Imiquad^®^ cream-treated mice on day 0. Image B shows signs of evident transformation and erythema in psoriatic mice treated with saline on day 7. Image D shows no irritation or erythema in psoriatic mice treated with optimized transferosomes on day 7.

No change in the skin conditions or irritation was recorded in animals treated with the optimized formulation (formulation DDT-10) (Figure 13). Results from the visual assessment demonstrated no irritation scores (Table 7). Therefore, the formulated berberine HCl and diacerein-loaded transferosomes can be recommended as safe for topical application.

**Table 7.** Results of erythema scores after application of normal saline and optimized transferosomes (formulation DDT-10) to the depilated dorsal skin of imiquimod-induced psoriatic BALB/c mice.

Day	Reference (normal saline)	Optimized formulation (formulation DDT-10)
1	0	0
2	0	0
3	1	1
4	2	1
5	3	0
6	4	0
7	4	0

### Stability study

**Figure 14.**
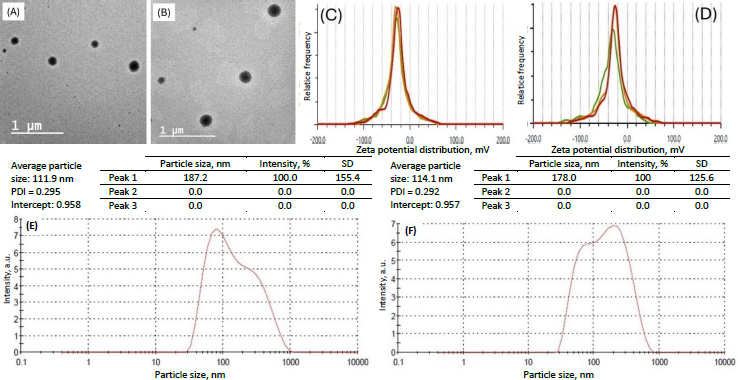
TEM images A and B, zeta potential results C and D, particle size distribution E and F of optimized transferosomes (formulation DDT-10) stored at 4±1 °C and 25±2 °C/ 60±5 % RH for 3 months, respectively.

Optimized transferosomes (formulation DDT-10) did not significantly change their physical appearance. No change in morphology (Figure 14A and B) or vesicle size (Figure 14E and F) was recorded. ζ potentials were within the range of ±30 mV, indicating the stable nature of the formulation stored at 4 °C (-25.4±1.8 mV) and 25±2 °C (-27.3±0.4 mV) for 3 months (Figure 14C and D, respectively).

The entrapment efficiency of the optimized formulation stored at 4 °C for 3 months was found to be 88.86±1.3 and 89.89±1.2 %, respectively, for berberine HCl and diacerein. The entrapment efficiency of the optimized formulation stored at 25±2 °C for 3 months was found to be 88.11±1.4 and 89.06±0.6 %, respectively, for berberine HCl and diacerein. Results indicated no significant leaching of the drug from the vesicles at both storage conditions.

## Conclusions

This communication reported formulation and optimization of berberine HCl and diacerein-loaded dual-delivery transferosomes. Transferosomes were successfully formulated using the thin film hydration method. The optimized transferosomes had particle size of 110.90±2.8 nm along with high entrapment efficiency (89.50±1.5 of berberine HCl and 91.23±1.8 % of diacerein) and outstanding release profile (82.09±0.81 and 85.02±3.81 % release of berberine HCl and diacerein, respectively, after 24 h), which makes them most suitable for skin penetration. The nonlinear quadratic polynomial model indicated the validity of the developed mathematical model, which can be used to produce dual delivery transferosomes with desired properties. The transdermal flux of the optimized formulation was 0.0224 μg cm^-2^ h^-1^ (permeation coefficient of 2.24 cm h^-1^) and 0.0462 μg cm^-2^ h^-1^ (permeation coefficient of 4.62 cm h^-1^), respectively, for berberine HCl and diacerein. Raman analysis of treated pig ear skin revealed that the transferosomes permeated the skin. No change in the skin condition or irritation was observed in BALB/c mice. The formulation was found to be stable at 4 and 25±2 °C/60±5 % RH for 3 months. The preliminary results of this study suggest that the phosphatidylcholine and sodium deoxycholate-based transferosomes can open a new avenue for potential topical co-delivery of berberine HCl and diacerein for psoriasis therapy.
